# Study on the Deterioration Mechanism of Magnesium Oxychloride Cement under an Alkaline Environment

**DOI:** 10.3390/ma16175924

**Published:** 2023-08-30

**Authors:** Lingyun An, Chenggong Chang, Fengyun Yan, Jianhong Peng

**Affiliations:** 1 Qinghai Provincial Key Laboratory of Nanomaterials and Technology, College of Physics and Electronic Information Engineering, Qinghai Minzu University, Xining 810007, China; anlingyun0825@126.com (L.A.); pjhhj@sohu.com (J.P.); 2 State Key Laboratory of Advanced Processing and Recycling of Non-Ferrous Metals, Lanzhou University of Technology, Lanzhou 730050, China; 3 Key Laboratory of Comprehensive and Highly Efficient Utilization of Salt Lake Resources, Qinghai Institute of Salt Lake, Chinese Academy of Sciences, Xining 810008, China; 4 Key Laboratory of Salt Lake Resources Chemistry of Qinghai Province, Xining 810008, China

**Keywords:** magnesium oxychloride cement, alkaline environment, microstructure, compressive strength, deterioration mechanism

## Abstract

The deterioration process and deterioration mechanism of magnesium oxychloride cement (MOC) in an alkaline environment were studied using a scanning electron microscope (SEM), an X-ray diffractometer (XRD), a Fourier transform infrared spectrometer (FT-IR) and a micro-electro-hydraulic servo pressure testing machine to investigate the effects of soaking time in 10 wt.% NaOH solution on the macro- and micro-morphology, phase composition and compressive strength of MOC samples. The results show that the deterioration of MOC samples under an alkaline environment is mainly caused by the alkaline environment providing more OH^−^ ions, which can react with 5Mg(OH)_2_·MgCl_2_·8H_2_O (P 5) in the sample. The resulting reaction gives rise to a faster decomposition of 5Mg(OH)_2_·MgCl_2_·8H_2_O (P 5) and a substantial reduction in the strength of the sample, and finally leads to a gradual deterioration of MOC samples. Meanwhile, immersion time exhibits a significant effect on MOC samples. The extension of immersion time coincides with more OH^−^ ions entering the sample, and the greater presence of OH ions increases the likelihood that more P 5 will produce a hydrolysis reaction, further resulting in the increased deterioration of the sample. After soaking for 6 h in alkaline media, the main phase composition of the surface layer of an MOC sample changes to MgO and Mg(OH)_2_, and its microscopic morphology is also dominated by round sheets, giving rise to a sharp decrease in its compressive strength (52.2%). When the immersion time is prolonged to 72 h, OH^−^ ions have already immersed into the inner core of the sample, causing the disappearance of P 5 from the whole sample. At the same time, both the surface and inner core of the sample exhibit a disc-shaped morphology, and chalking phenomena also appear on the surface of the sample. This reduces the compressive strength of the sample to 13.5 MPa, only 20% of its compressive strength in water. The compressive strength of the sample after 120 h of immersion is as low as 8.6 MPa, which is lower than that of the sample dipped in water for 21 days (9.5 MPa). As a result, the MOC samples studied in alkaline environments exhibit a faster deterioration rate, mainly because of a faster hydrolysis reaction by P 5, caused by more OH^−^ ions.

## 1. Introduction

Qarhan Salt Lake is a large, comprehensive deposit of potassium and magnesium salts located in the middle and eastern part of Qaidam Basin in Qinghai Province, China. It is mainly composed of liquid mixed with some solids, where the stock of KCl is 2.9862 × 10^8^ t and the content of MgCl_2_ is 38.5622 × 10^8^ t [1]. Since 1957, scientists have carried out a large number of studies on the development of potash fertilizer and its applications. Now, Qarhan Salt Lake is China’s largest potash production base. However, there are significant quantities of bischofite accumulating in Qarhan Salt Lake after years of potassium extraction and production, forming “magnesium damage.” This seriously affects the surrounding environment. At present, the exploiting the application of magnesium oxychloride cement (MOC) has become an ecological, economic, effective and novel way to solve the problem presented by large amounts of bischofite, in addition to using bischofite to produce metallic magnesium, magnesium oxide, magnesium hydroxide, magnesium compound whisker and other magnesium products [2,3].

MOC, a good adhesive material, is formed in the ternary system composed of MgO, MgCl_2_ and H_2_O, which is also called Sorel Cement, and was found by Sorel et al. in 1867 [4,5,6]. MOC possesses a series of advantages including high strength, fast hardening, good elasticity, high surface gloss and a simple preparation process [7]. Today, it is described as an ecological and environmentally friendly material due to its energy-saving, fire prevention, heat insulation and sound insulation properties [8]. Additionally, MOC has a low carbon footprint. Ondřej Jankovský et al. [9] have pointed out that both 3Mg(OH)_2_‧MgCl_2_·8H_2_O (Phase 3) and 5Mg(OH)_2_·MgCl_2_·8H_2_O (Phase 5) in MOC possess the capacity for fast mineral carbonation and have high maximum theoretical values of CO_2_ uptake capacity. Therefore, MOC is widely used in door core panels, building partition panels, arts and crafts, and transportation boxes.

In recent years, the research on MOC materials has mainly focused on the development of raw materials, performance optimization and functional applications, which has led to a wealth of research achievements [10,11,12,13]. However, there are few studies on the durability of MOC materials, which is not only attributable to their own composition and structure, but also to the external service environment. Different service environments determine the deterioration process and deterioration rate of materials. Furthermore, research on the deterioration mechanism of materials under different service environments can provide engineering experience that enhances the design and optimization of materials. This can significantly reduce the economic cost of the materials while extending their life. For example, Hadi H. Edan et al. [14] studied the durability of eco-efficient, self-compacting concrete (SCC) using partially contained waste walnut shell particles as fine aggregate, and found that the SCC mixture containing 40% WS possessed the lowest compressive strength of 23.7 MPa due to having more empty space and less compactness. Meanwhile, the density, compressive and splitting tensile strengths of all SCC mixes decreased after periods of exposure to both H_2_SO_4_ and MgSO_4_ solution attacks. Nibras Y. Alani et al. [15] investigated the performance of SCC containing nano clay at elevated temperatures and exposed to a MgSO_4_ attack, and pointed out that the nano clay incorporating SCC specimens possessed higher durability properties. With respect to MOC, so far, the effects of a high salt environment, high heat environment, dry-wet cyclic environment and long-term indoor environment on the deterioration properties of MOC materials have also been reported [16,17,18,19]. However, if MOC materials are exposed to an environment with chemical substances, such as the alkaline environment of a production workshop, they may be damaged by chemical erosion. Yet, there are few reports on the corrosion damage to MOC that may be caused by an alkaline environment. Therefore, it is necessary to study the deterioration performance and process of MOC in an alkaline environment.

In this paper, based on the full immersion experiment, SEM, XRD, FT-IR and micro-electro-hydraulic servo pressure testing machines were used to study the evolutions of the macro- and micro-morphology, quality, phase composition and compressive strength of MOC samples in 10 wt.% NaOH solution with immersion time. Simultaneously, the deterioration process and deterioration mechanism of MOC samples under an alkaline environment were revealed. This would enrich and develop the durability theory concerning MOC materials in special environments.

## 2. Materials and Experiment

### 2.1. Principal Raw Materials

Bischofite, a kind of white crystal, is the by-product generated through the extraction of potassium, which originates from Golmu in Qinghai Province. Its chemical composition is mainly MgCl_2_·6H_2_O, as listed in Table 1.

Light-burned magnesia comes from the calcination of magnesite produced in Haicheng in Liaoning Province. Its activity is 50.51% and its chemical composition is mainly MgO, as shown in Table 2.

### 2.2. Preparation of MOC Samples

Firstly, 23.5 wt.% MgCl_2_ aqueous solution was prepared. Secondly, MOC samples were fabricated on the basis of a 7.2:1:16 molar ratio of active MgO, MgCl_2_ and H_2_O. Their detailed preparation process was as follows: light-burned magnesia powders and pre-prepared 23.5 wt.% MgCl_2_ aqueous solution were mixed evenly via constant stirring to form a slurry, which was injected into a 20 mm × 20 mm × 20 mm cube mold to harden in an indoor environment (at a temperature of 21 ± 1 °C, with 40 ± 1% humidity). After 24 h, the MOC samples were taken out from the mold, and continued to cure for 28 days under an indoor environment. These were the MOC slurry samples, and were labeled as 0 h.

### 2.3. Immersion Experiment

Firstly, a NaOH aqueous solution with a mass fraction of 10% was prepared, and then the MOC slurry samples were completely soaked in this alkaline solution which was renewed every hour, according to the literature [20]. The samples were taken out from the alkaline media after 1 h, 6 h, 9 h and 12 h, respectively. Meanwhile, the weight and compressive pressure of the samples were tested immediately. The compressive strength of the MOC samples before and after immersion were also separately calculated according to Equation (1). At the same time, their standard deviations were obtained.

(1) $$P=\frac{F}{4}\times 10$$

where *F* is the pressure, viz, the average value of 6 samples, and its unit is KN; 4 is the sample area, and its unit is cm^2^; *P* is the ultimate compressive strength of the sample, given in MPa.

### 2.4. Characterization

A CP513 electronic balance was used to evaluate the weight of the MOC sample, in which 6 parallel specimens were taken, and each specimen was weighed 5 times. The obtained average value was namely the mass of the samples, and the corresponding standard deviations were achieved. Field emission scanning electron microscopy (FE-SEM, SU8010, Hitachi High-Tech, Tokyo, Japan) was used to study the micro-morphology of the sample, and the acceleration voltage was 2.0 kV. Before the analysis, the surface of the sample was sprayed with gold. The phase composition of the sample was detected using an X-ray diffractometer (XRD, D8 Discover, Bruker, Karlsruhe, Germany) with a copper target as the anode. The scanning angle was 5–80° and the scanning step was 0.02°. A Fourier transform infrared spectrometer (FT-IR, Nexus, Thermo-Nicolet, Madison, NY, USA) was applied to test the characteristic peak of the phase composition with a resolution of 4 cm^−1^ at a scan range from 4000 to 400 cm^−1^. A micro-electro-hydraulic servo pressure testing machine (model HYE-300B-D, Beijing sanyuweiye testing machine Co., Ltd., Beijing, China) was used to test the compressive pressure of the sample and calculate the compressive strength of the sample under the condition of a loading rate of 2.4 KN/s, according to the Chinese national standard GB/T 17671-1999 [21].

## 3. Results

### 3.1. Macroscopic Morphology Analyses

**Figure 1:**
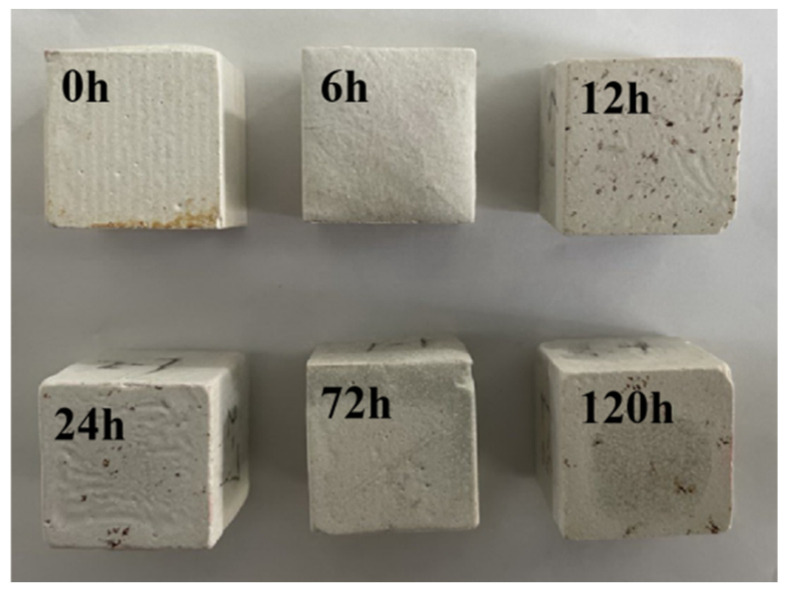
Macroscopic photos of MOC samples soaked in alkaline solution for different periods of time.

Macroscopic photos of MOC samples soaked in 10 wt.% NaOH solution for different periods of time are shown in Figure 1. It can be seen from Figure 1 that the surface of the MOC slurry sample (0 h) is milky white. With the extension of the immersion time from 6 h to 120 h, the color of the surface of the MOC sample gradually darkens, but the sample still remains intact, and no apparent cracks can be observed. Additionally, there is no serious damage or any falling-off phenomenon occurring at the corners.

### 3.2. Weight Analyses

**Figure 2:**
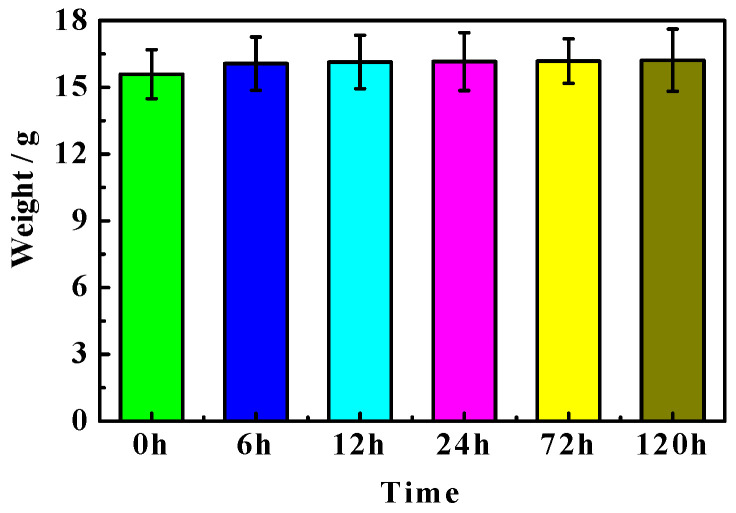
Weight of MOC samples immersed in alkaline solution for different periods of time.

Figure 2 presents the weight of the MOC samples dipped in 10 wt.% NaOH media for different periods of time. As can be seen from Figure 2, the quality of the MOC sample before immersion (0 h) is 15.59 g. With prolonging the soaking time, the MOC sample becomes slightly heavy. See 6 h, 12 h, 24 h, 72 h and 120 h presented in Figure 2. This may be due to the fact that the increased content in the sample is greater than the amount that is lost from the sample.

### 3.3. Compressive Strength Analyses

**Figure 3:**
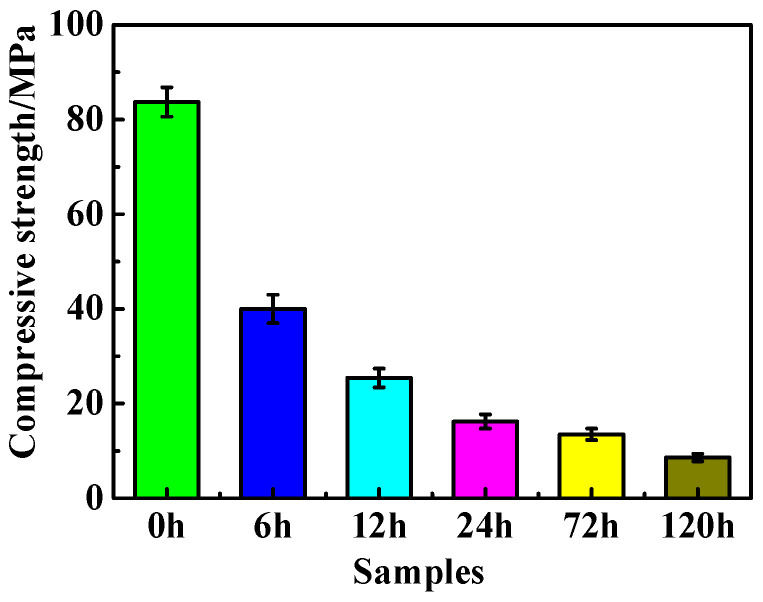
Compressive strength of MOC samples dipped in alkaline solution for different times.

The relationship between the compressive strength of the MOC samples and the immersion time in 10 wt.% NaOH solution is presented in Figure 3. It can be observed from Figure 3 that the compressive strength of the MOC slurry sample that was steeped for 0 h is 83.7 MPa, but with the extension of soaking time, the compressive strength of the MOC sample gradually decreases, and the reduced extent is much larger. Compared with the compressive strength of the MOC sample before soaking (0 h), the compressive strength of the sample after immersion for 6 h decreased by 52.2%, and it declined by nearly 70% after 12 h. When the immersion time was further extended to 72 h, the compressive strength was only 16.1% of what it was before immersion (0 h). After immersing for 120 h, the compressive strength of the sample is as low as 8.6 MPa. These data indicate that the alkaline environment has a great influence on the mechanical properties of the MOC samples.

### 3.4. Microscopic Morphology Analyses

**Figure 4:**
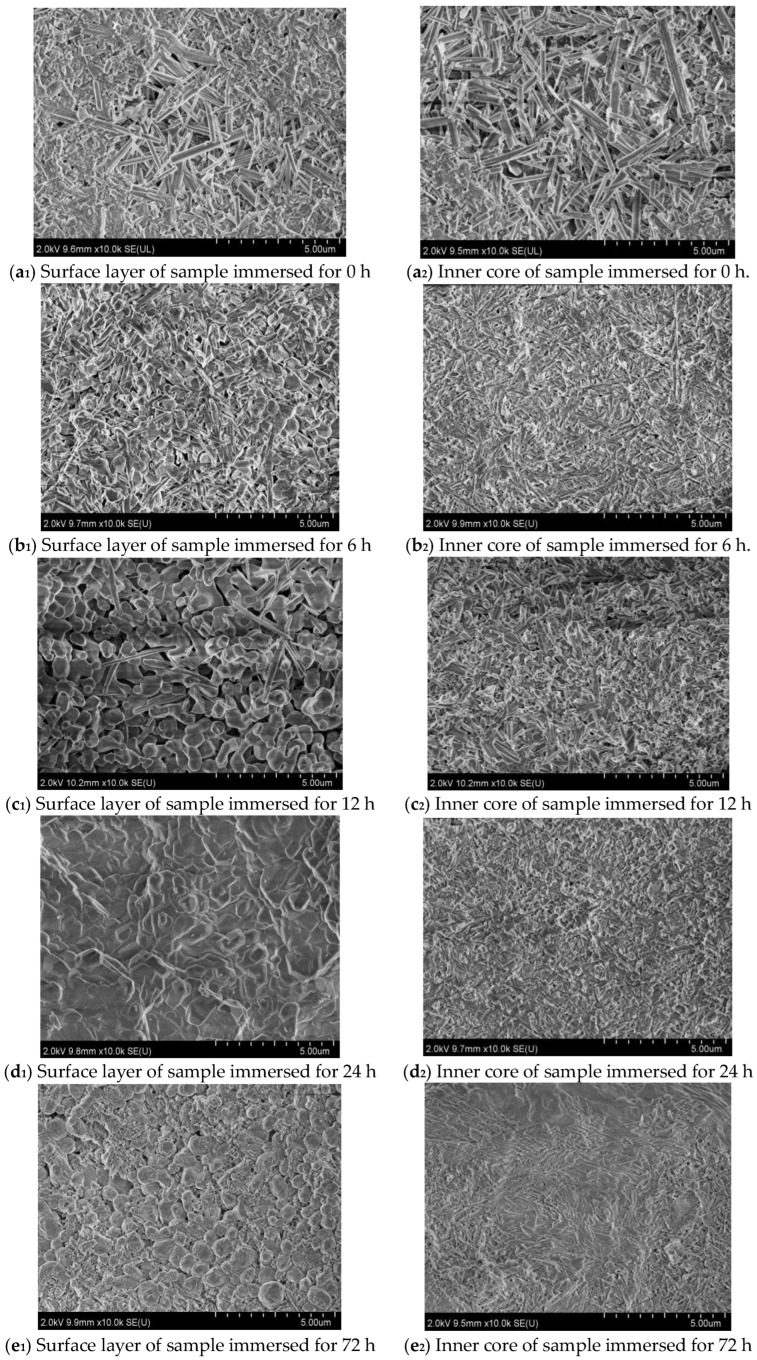

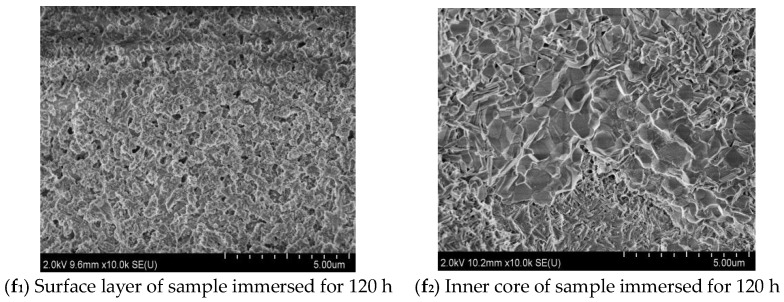
Microscopic morphologies of the surface layer and inner core of MOC samples soaked in alkaline solution for different periods of time.

Figure 4 displays the micro-morphology of the surface and inner core of the MOC samples steeped in 10 wt.% NaOH solution for different periods of time. It can be seen from Figure 4 that with the extension of soaking time, an obvious alteration in the microscopic morphology of the surface layer and inner core of the sample takes place. The surface and inner core of the MOC slurry sample (0 h) both exhibit a gel-like and rod-like morphology; see Figure 4(a_1_,a_2_). They interleave with each other and are relatively compact, which is conducive to improving the mechanical properties of the MOC sample. After soaking for 6 h, a round-sheet morphology appears on the surface of the sample (Figure 4(b_1_,b_2_)). This morphology is more obvious when the immersion time is extended to 24 h, but the inner core is still gel-like and rod-like, as presented in Figure 4(d_1_,d_2_). It can be observed from Figure 4(e_1_,e_2_) that after soaking in alkaline solution for 72 h, the surface layer and inner core of the sample both possess round-sheet morphologies, and the surface layer also presents a granular morphology, indicating that the surface layer of the sample appears affected by a powder phenomenon after the immersion of 72 h. At the same time, the NaOH solution has already penetrated into the inside of the sample. From Figure 4(f_1_,f_2_), it can be observed that after 120 h, the chalking phenomenon on the surface of the sample is more serious, and the round-sheet morphology of the inner core is more obvious, which also suggests that the deterioration of the sample is even worse.

### 3.5. Phase Composition Analyses

**Figure 5:**
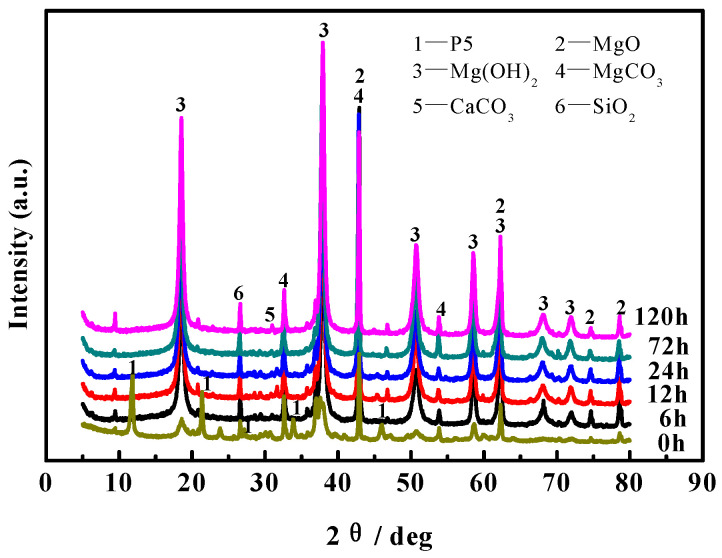
XRD patterns for the surface layer of MOC samples after being dipped in the alkaline solution for different periods of time.

According to the literature [22,23], MOC is mainly composed of 5Mg(OH)_2_·MgCl_2_·8H_2_O (abbreviated as 5·1·8 phase, P 5), Mg(OH)_2_, MgCO_3_, CaCO_3_, MgO, SiO_2_ and other components. Figure 5 shows XRD patterns of the MOC samples dipped in 10 wt.% NaOH solution for different periods of time. As can be seen from Figure 5, compared with the MOC slurry samples (0 h), the diffraction peaks of P 5 in the surface layer of the samples disappear after immersion. Moreover with the extension of soaking time, the diffraction peaks of MgO slightly decrease, yet the diffraction peaks of Mg(OH)_2_ gradually increase. This indicates that after dipping the MOC samples into the alkaline media, P 5 in the surface layer fades away and the content of MgO slightly declines; however, the content of Mg(OH)_2_ is augmented.

**Figure 6:**
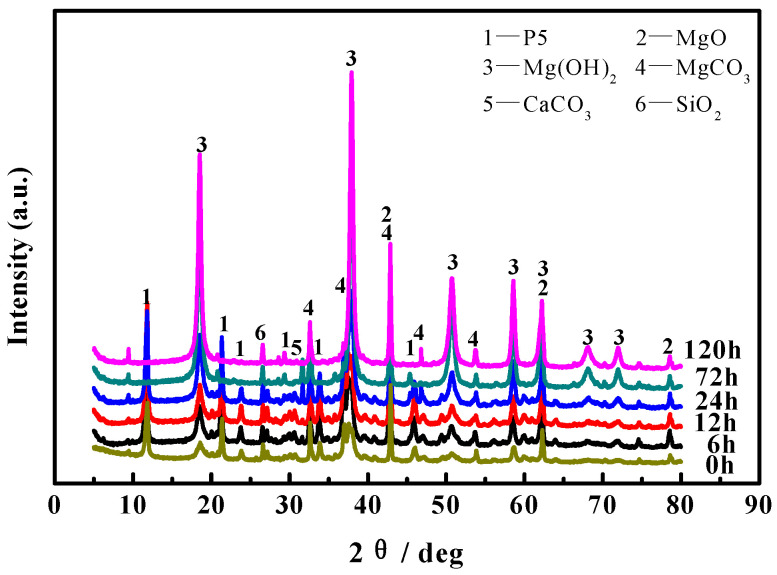
XRD patterns for the inner core of MOC samples after being immersed in alkaline solution for different periods of time.

XRD patterns with respect to the inner cores of MOC samples soaked in 10 wt.% NaOH solution for different periods of time are presented in Figure 6. It can be seen from Figure 6 that compared with the samples before immersion (0 h), the diffraction peaks of P 5 that existed in the inner core of the MOC sample decrease slightly after an immersion time of 6 h, and they almost disappear after 72 h. This indicates that the NaOH solution has already penetrated into the inner core of the sample after being dipped in 10 wt.% NaOH solution for 72 h, resulting in an obvious change in the main phase composition of the inner core of the sample. This is consistent with the results in Figure 4.

### 3.6. FT-IR Spectra Analyses

**Figure 7:**
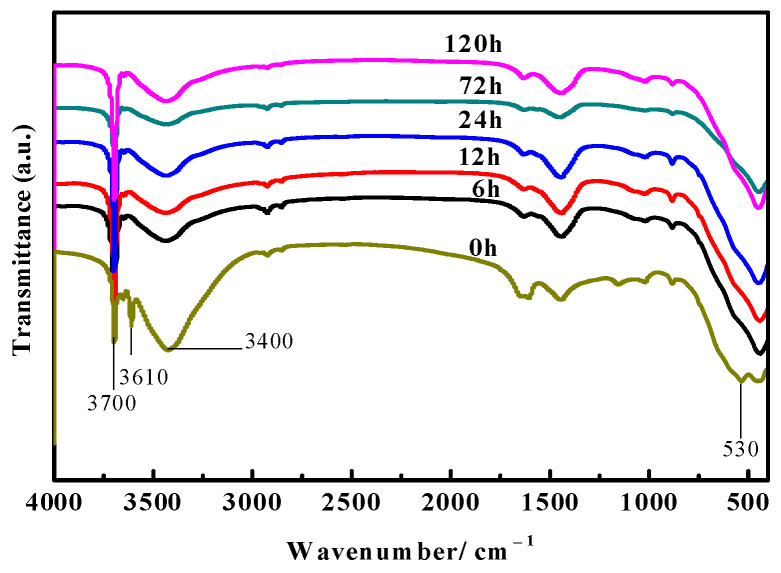
FT-IR spectra of the surface layer of samples steeped in alkaline solution for different times.

Figure 7 displays the FT-IR spectra of the surface layer of the MOC samples soaked in 10 wt.% NaOH solution for different periods of time. Researchers [24,25] have pointed out that the stretching vibration absorption band of the Mg-O bond is near 530 cm^−1^, which belongs to the characteristic peak of MgO. Between 3330 cm^−1^ and 3500 cm^−1^ is the characteristic peak of the vibration of the coordinated water molecules. The sharp absorption peaks at 3670 cm^−1^ and 3610 cm^−1^ are the non-aqueous hydroxyl (−OH) stretching vibration peak superimposed on the crystal water, which are the characteristic peaks of P 5. The difference in peak intensity at 3700 cm^−1^ indicates that the content of Mg(OH)_2_ is diverse. As can be observed from Figure 7, the characteristic peak near 3610 cm^−1^ disappears after soaking treatment in comparison with the MOC slurry sample (0 h), indicating that there is no P 5 present in the surface layer of the sample. This effect is produced when the samples suffer from erosion caused by the alkaline solution. At the same time, the characteristic peak near 3700 cm^−1^ gradually increases, while the characteristic peak near 530 cm^−1^ slightly decreases, suggesting that immersion treatment in alkaline media increases the content of Mg(OH)_2_ in the surface layer of the sample, yet decreases the content of MgO somewhat, which is in accord with the XRD analysis in Figure 5.

**Figure 8:**
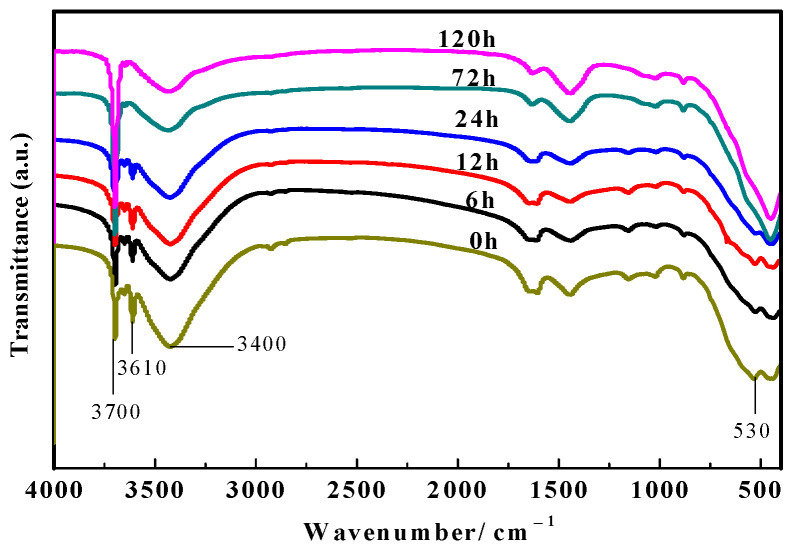
FT-IR spectra of inner cores of samples dipped in alkaline solution for different periods of time.

Figure 8 presents the FT-IR spectra of the inner cores of the MOC samples soaked in 10 wt.% NaOH solution for different periods of time. As can be seen from Figure 8, compared with the sample dipped in the alkaline media for 0 h, the characteristic peak near 3610 cm^−1^ disappears after an immersion time of 72 h, indicating that the P 5 in the inner core of the sample vanishes. In addition, the characteristic peak at 3700 cm^−1^ gradually increases with the extension of soaking time, but the characteristic peak near 530 cm^−1^ gradually weakens, suggesting that prolonging the immersion time in the alkaline solution increases the amount of Mg(OH)_2_ in the inner core of the sample, yet decreases MgO content slightly, which is consistent with the results of XRD analysis in the inner core shown in Figure 6.

## 4. Discussion

Magnesium oxychloride cement is an air-hardening, inorganic cementitious material which is prepared by mixing light-burned magnesia powders into MgCl_2_ solution to form a paste, which is then injected into the mold to harden in the air [26]. In general, the main hydration products of magnesium oxychloride cement are magnesium hydroxide (Mg(OH)_2_) and magnesium oxychloride, including 3Mg(OH)_2_·MgCl_2_·8H_2_O (abbreviated as 3·1·8 phase, P 3) and 5Mg(OH)_2_·MgCl_2_·8H_2_O (abbreviated as 5·1·8 phase, P 5), see Equations (2)–(4) [27]. Additionally, in this study, since the molar ratio of active MgO/MgCl_2_ is greater than 6, the MOC slurry samples are mainly composed of P 5, Mg(OH)_2_ and unreacted active MgO, as shown in the XRD patterns of samples (0 h) in Figure 5 and Figure 6.

MgO + H_2_O→Gel Mg(OH)_2_ (2)

3MgO + MgCl_2_ + 11 H_2_O→3Mg(OH)_2_·MgCl_2_·8H_2_O (3)

5MgO + MgCl_2_ + 13 H_2_O→5Mg(OH)_2_·MgCl_2_·8H_2_O (4)

**Figure 9:**
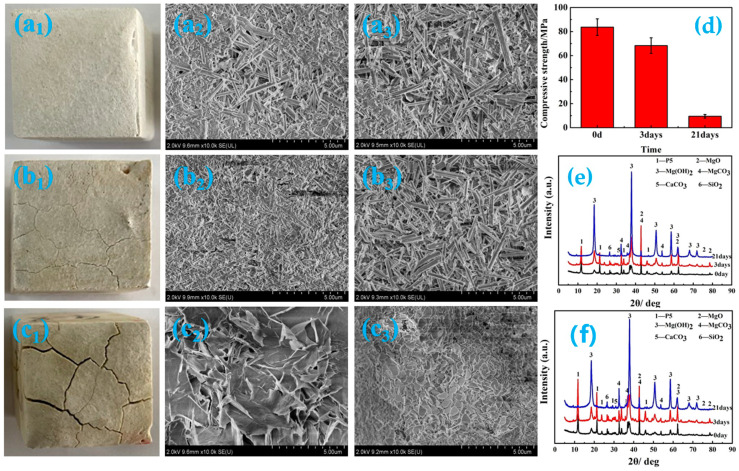
Macroscopic morphology (**a_1_**,**b_1_**,**c_1_**), micro-morphologies of surface (**a_2_**,**b_2_**,**c_2_**) and inner core (**a_3_**,**b_3_**,**c_3_**), compressive strength (**d**) and XRD patterns of surface layer (**e**) and inner core (**f**) for MOC samples soaked in water for different durations: (**a_1_**–**a_3_**) 0 days, (**b_1_**–**b_3_**) 3 days, (**c_1_**–**c_3_**) 21 days.

When the MOC sample is used in water, it will be damaged mainly by the OH^−^ ions that form the weak ionization of H_2_O, which will make P 5 in the sample hydrolyze, and cause the active MgO to be hydrated (see Formulas (5) and (6)), and finally result in the deterioration of the sample, as shown in Figure 9. Figure 9 displays the macroscopic and microscopic morphology, compressive strength and phase composition of the MOC samples soaked in water for different periods of time. As can be observed from Figure 9, when the MOC sample is immersed in water for 3 days (72 h), there are macroscopic cracks on the surface of the sample. In the meantime, the amounts of P 5 and MgO decrease, yet Mg(OH)_2_ content increases in the surface layers of the samples. Furthermore, the microscopic morphology of the surface layers and inner cores of the samples are still in a gel state and needle-rod shape, and the compressive strength is 68.3 MPa. When the soaking time is further extended to 21 days, the macroscopic cracks become wide and long, and crisscross each other. The P 5 and MgO in the entire sample almost disappear, and the Mg(OH)_2_ content increases sharply. The microscopic morphology of the sample surface becomes flaky, and the inner core also exhibits an obvious sheet morphology. As a result, the compressive strength decreases to 9.5 MPa. These results indicate that when the MOC sample is dipped in water for 3 days, only the outer layer is slightly degraded, resulting in a decrease in the mechanical properties of the sample. However, when the MOC sample is soaked for 21 days, the water molecules completely immerse the inside of the sample, causing a battery of changes comprising the macro-morphology, the micro-morphology of the surface layer and inner cores, along with the main phase composition. These result in a significant decrease in the mechanical properties of the sample.

Mg(OH)_2_·MgCl_2_·8H_2_O + OH^−^→5Mg(OH)_2_ + MgCl_2_(Loss) (5)

active MgO + OH^−^→Mg(OH)_2_ (6)

However, when MOC samples are applied in alkaline environments, they are eroded by OH^−^ ions which come from not only the weak ionization of H_2_O, but also the strong ionization of NaOH. This results in a higher content of OH^−^ ions in the solution and a faster hydrolysis reaction for P 5 (Equation (5)). While P 5 is the main strength phase, its decrease in content inevitably leads to the reduction in the mechanical properties of the sample. Therefore, when the MOC sample is soaked in 10 wt.% NaOH solution for 72 h, P 5 almost disappears from the entire sample. The surface and inner core of the sample exhibit a circular sheet morphology, and the surface layer also exhibits the phenomenon of micronization. The compressive strength of the sample is reduced to 13.5 MPa, which is only 20% of that in water. After steeping for 120 h (5 days), the compressive strength of the sample is reduced to 8.6 MPa, which is lower than when it has been immersed in water for 21 days (9.5 MPa).

In addition, MgO is an alkaline oxide, and the alkaline environment inhibits the hydration reaction of MgO. As a result, the MgO content in the MOC samples only decreases slightly with the extension of soaking time in 10 wt.% NaOH solution. The volume of Mg(OH)_2_ is larger than that of MgO, but it is much smaller than that of P 5; for this reason, the overall volume of MOC material does not expand significantly with the extension of soaking time in alkaline solution. Therefore, when the MOC sample is dipped in alkaline solution for 72 h, although its mechanical properties are greatly reduced, the sample’s surface is still intact, which differs from those samples soaked in water for 3 days (72 h). After an immersion time of 3 h in water, the sample possesses a relatively high compressive strength, but small cracks appear on the surface of the sample.

**Figure 10:**
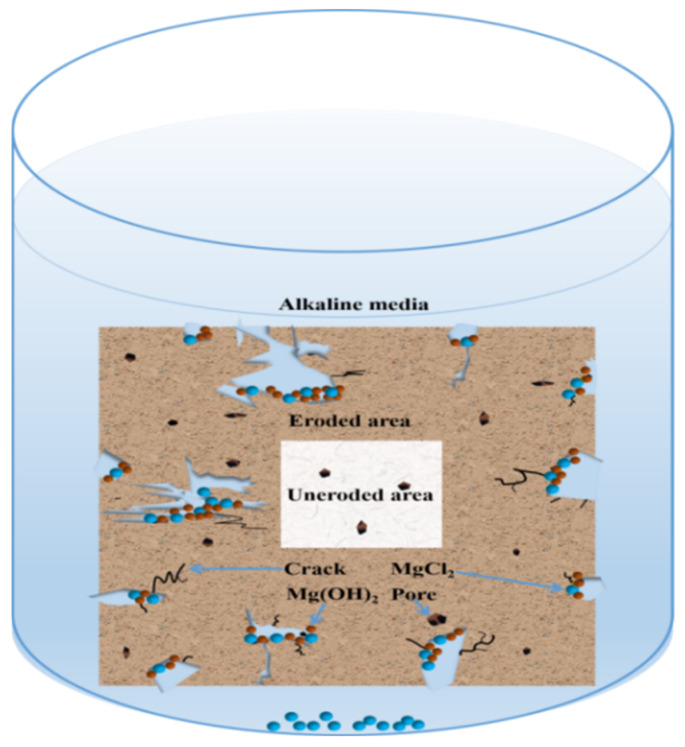
Schematic diagram of deterioration mechanism of MOC sample in the alkaline environment.

Obviously, the degradation mechanism of the MOC samples in an alkaline environment is unlike that demonstrated by water. The former is mainly a product of the alkaline environment which provides more OH^−^ ions, which can then react with P 5 in the sample, leading to a faster decomposition of the main strength phase P 5 in the sample, and further results in a substantial reduction in the strength of the sample and its gradual deterioration. The possible mechanism is shown in Figure 10.

## 5. Conclusions

1. The deterioration of the MOC sample in an alkaline environment is mainly due to the fact that the alkaline environment provides more OH^−^ ions, which can react with P 5 in the sample, giving rise to a faster decomposition of the main strength phase P 5 in the sample. This further results in a substantial reduction in the strength of the sample and its gradual deterioration.
2. Immersion time also exhibits a significant effect on the MOC sample. With the prolongation of the immersion time of the sample in an alkaline medium, a greater number of OH^−^ ions can enter the sample, which causes increasing P 5 to undergo a hydrolysis reaction, and results in more serious deterioration for the sample. The MOC slurry sample is mainly composed of P 5, Mg(OH)_2_ and MgO. Meanwhile, it possesses a gel-like and a needle-like micro-morphology, which are interleaved with each other and relatively compact. As a result, the MOC slurry sample has a high compressive strength of 83.7 MPa. After soaking for 6 h in an alkaline medium, the main phase composition of the surface layer of the MOC sample changes to MgO and Mg(OH)_2_, and the microscopic morphology is also dominated by a round-sheet, resulting in a sharp decrease in compressive strength (52.2%). When the immersion time is extended to 72 h, OH^−^ ions have already been immersed into the inner core of the sample, giving rise to the disappearance of P 5 from the entire sample. Meanwhile, both the surface layer and inner core of the sample exhibit a round-sheet morphology, and a powdering phenomenon also appears on the surface layer. Therefore, the compressive strength of the sample is reduced to 13.5 MPa, which is only 20% of its strength when dipped into water. After immersion for 120 h (3 days), the compressive strength of the sample is as low as 8.6 MPa, which is weaker than that when it has been steeped in water for 21 days (9.5 MPa), presenting a faster deterioration rate.

## Figures and Tables

**Table 1:** Chemical composition of bischofite.

Composition	MgCl_2_	NaCl	MgSO_4_	KCl	CaCl_2_	Water-Insoluble Matter	H_2_O
Content (wt.%)	44.90	0.13	0.06	0.01	0.03	0.27	54.6

**Table 2:** Chemical composition/(wt.%) of light burned magnesia.

Composition	MgO	MgCO_3_	CaCO_3_	Free CaO	Acid-Insoluble Matter
Content (wt.%)	69.52	19.80	1.34	0.38	8.41

## Data Availability

The data presented in this study are available upon request from the corresponding author and the first author.
